# Association of periodontal disease treatment with mortality in patients with dementia: a population-based retrospective cohort study (2002–2018)

**DOI:** 10.1038/s41598-024-55272-6

**Published:** 2024-03-04

**Authors:** Han-A Cho, Bo-Ra Kim, Hosung Shin

**Affiliations:** 1 Department of Dental Hygiene, Shinhan University, Uijeongbu-si, Gyeonggi-do 11644 Republic of Korea; 2 Department of Dental Hygiene, Daejeon Health Institute of Technology, Dong-gu, Daejeon Metropolitan City 34504 Republic of Korea; 3 Department of Social and Humanity in Dentistry, Wonkwang University School of Dentistry, 460 Iksan-daero, Iksan, North Jula 54538 Republic of Korea

**Keywords:** Health care, Risk factors

## Abstract

Dementia is one of the leading causes of death worldwide. In this study, we analyzed the association of periodontal treatment with the risk of death in patients with dementia. The analyzed data were obtained by linking the National Health Insurance Corporation claims data between 2002 and 2018 to the Statistics Korea death registry. In total, 1,131,406 patients with dementia aged ≥ 65 years had undergone dental treatment during the study period. Time-dependent Cox proportional hazards model was performed. The mortality rate was approximately 10% among the patients with dementia. The 17-years cumulative survival rates for patients who received periodontal treatment and their untreated counterparts were 83.5% and 71.5%, respectively. The crude hazard ratio of the periodontal group was approximately twice as high as that of the non-periodontal group (1.99; *P* < 0.001). Furthermore, in the regression model that was adjusted for socio-demographic variables and systematic chronic diseases, the risk of death in the non-periodontal group was approximately 1.83 times higher than that of the periodontal group (*P* < 0.00). These findings suggest that preventive periodontal treatment may decrease mortality risk in older people with dementia.

## Introduction

The severity of dementia is directly associated with high mortality rates^1^. In 2016, dementia-associated mortality increased more than twice compared with that in 1990^2^ and exceeded 55 million in 2021, with an annual increase of 10 million deaths^3^. In the Global Burden of Diseases, Injuries, and Risk Factors Study, Alzheimer’s disease and other types of dementia were ranked fourth in the disability-adjusted life years of people over the age of 75 years^4^.

Dementia-related studies are being conducted in several areas, such as physical, psychological, social, and economic fields of study. Alzheimer’s disease is associated with physical and systemic manifestations. Individual genetic and environmental factors, such as mid-life obesity, insulin resistance, and inflammatory processes, are risk factors for Alzheimer’s disease^5^. Antihypertensives, statins^6^, behavior management, and cognitive stimulation^7^ have been reported as effective treatment approaches for dementia. According to the cost-of-illness studies related to dementia, the overall main cost drivers of dementia-related care were informal costs associated with home-based long-term care and nursing home expenditures rather than direct medical costs^8^. Individual characteristics, such as old age and male sex, have been associated with increased severity of dementia and Alzheimer’s disease; functional impairment has also been reported as a factor linked with dementia-associated mortality^9^.

Several studies investigating the relationship between oral health and dementia reported that patients with dementia had fewer teeth, more carious teeth, worse oral hygiene, and poorer periodontal health than patients without dementia^10^. Martande et al.^11^ revealed that patients with Alzheimer’s disease had higher rates of poor oral health signs and symptoms in terms of gingival index, plaque index, probing depth, clinical attachment level, and percentage of bleeding sites. In addition, the periodontal condition worsened as Alzheimer’s disease progressed. Meanwhile, several studies have examined the impact of periodontal disease on dementia^11,12^. A study identified a main pathogen of chronic periodontitis in the brains of patients with Alzheimer’s disease and suggested that inhibiting toxic proteases released by the pathogens may be a potential treatment approach for dementia^13^. Scherer and Scherer^14^ reported that Alzheimer’s disease-associated mortality was related to edentulous or partially edentulous jaws. The authors claimed that periodontal disease treatments could substantially reduce the clinical, social, and financial burden of Alzheimer’s disease.

Many studies have focused on the association between dementia and oral health as well as dementia and mortality. However, previous studies have not directly linked periodontal treatment with dementia-related mortality. Only a recent study has examined the association between periodontal pathogens and Alzheimer’s disease-associated mortality; they reported that periodontal disease contributes to mortality in patients with dementia^15^ and suggested the possibility of improving the oral health of vulnerable elderly individuals through dental care. In this study, we aimed to analyze the association between periodontal treatment and survival rates in patients with dementia using population-based retrospective cohort data with up to 17 years of follow-up in South Korea.

## Results

### General characteristics of the participants

The mortality rate among the study participants was approximately 10% (Table 1). The percentage of patients with dementia who received periodontal treatments was 8.58%. In periodontal treatment classification, the percentage of patients with dementia who received SRP, subgingival curettage, and periodontal flap surgery were 8.33%, 1.07%, and 0.31%, respectively. In this study, 9.2% of the participants had chronic diseases, where cerebrovascular diseases constituted the highest proportion (approximately 5%) among all the chronic diseases. Among the study participants, the number of females was more than twice the number of males. The proportion of residents living in metropolitan areas and small- and medium-sized cities was almost similar; the residents living in rural areas constituted the lowest proportion of study participants.

**Table 1:** Characteristics of the study participants stratified by the dementia type.

Variables	Total (N = 1,131,406)	Alzheimer’s dementia (N = 985,555)	Vascular dementia (N = 145,851)	*P* value
	100 (%)	87.11 (%)	12.89 (%)	
Periodontal treatment experience^a^				
No	91.42	91.2	93.0	< 0.001
Yes	8.58	8.8	7.0	
Periodontal treatment classification				
SRP				
No	91.67	91.4	93.2	< 0.001
Yes	8.33	8.6	6.8	
Subgingival curettage				
No	98.93	98.9	99.0	< 0.001
Yes	1.07	1.1	1.0	
Periodontal flap surgery				
No	99.69	99.7	99.7	0.005
Yes	0.31	0.3	0.3	
Systematic diseases				
No	90.82	91.6	85.4	< 0.001
Ischemic heart diseases	0.51	0.5	0.6	
Cerebrovascular diseases	5.22	4.4	10.6	
Kidney disease	0.18	0.2	0.2	
Diabetes mellitus	3.27	3.3	3.2	
Sex				
Male	31.12	30.3	36.9	< 0.001
Female	68.88	69.7	63.1	
Health insurance				
Health insurance holders	87.78	87.8	87.9	0.259
Medical aid	12.22	12.2	12.1	
Residential areas				
Metropolitan areas	40.77	40.8	40.4	< 0.001
Small-/medium-sized cities	39.17	39.1	39.8	
Rural areas	20.06	20.1	19.8	
Death				
No	90.02	90.2	89.1	< 0.001
Yes	9.98	9.8	10.9	
Age	80.12 ± 6.83	81.50 ± 6.78	79.62 ± 6.89	< 0.001

Numerical value: mean ± standard deviation or n (%).

*SRP* scaling or root planning.

^a^Periodontal treatment experience: SRP or subgingival curettage or periodontal flap surgery.

Demographic distributions of the Alzheimer’s-related dementia (hereafter Alzheimer dementia) and vascular-related dementia (hereafter vascular dementia) groups were similar, except for sex and chronic disease status. Furthermore, the proportion of males and patients with chronic diseases was high in the vascular dementia group. In particular, the rate of cerebrovascular disease in the vascular dementia group was more than double the rate in the Alzheimer dementia group.

### Survival probability

**Figure 1:**
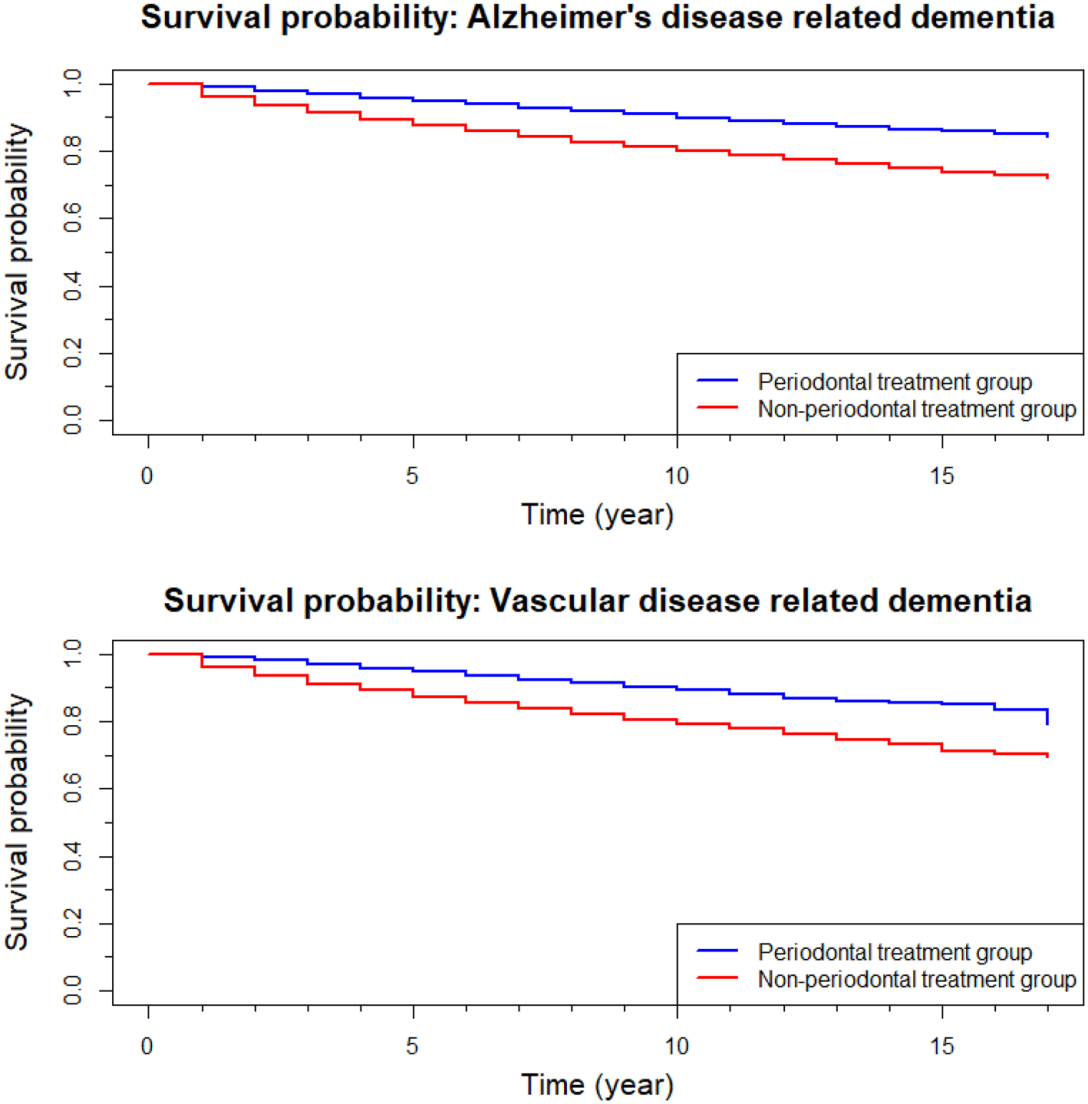
Survival probability of the patients receiving periodontal treatment. (**a**) Alzheimer’s disease-related dementia and (**b**) vascular disease-related dementia.

The 17-year cumulative survival rates in the periodontal treatment group and non-treatment group were 83.5% (95% confidence interval [CI] 0.822–0.849) and 71.5% (95% CI 0.712–0.718), respectively. The 17-years survival rate of participants with Alzheimer dementia who received periodontal treatment (84.2%; 95% CI 0.830–0.854) was higher than that of participants in the vascular dementia group (79.1%; 95% CI 0.728–0.858; Fig. 1).

### Time-dependent cox proportional hazard model

Hazard ratio (HR) according to the periodontal treatment was analyzed by considering the time-dependent characteristics of chronic disease variables. Participants who did not receive periodontal treatments had an approximately 1.99 times higher risk of death (Model I, crude HR) than their counterparts who received treatments. In contrast, in the model adjusted for socio-demographic characteristics (Model II), participants who did not receive periodontal treatments had an approximately 1.83 times higher risk of death (adjusted HR) compared with participants who received periodontal treatments.

Compared with participants without chronic diseases, the HRs of patients with chronic diseases were 1.4, 1.07, 3.25, and 1.54 times higher for those with ischemic heart diseases, cerebrovascular diseases, kidney diseases, and diabetes mellitus, respectively. In addition, the HRs were high in males, beneficiaries of medical aid, and residents of metropolitan areas (Table 2).

**Table 2:** The time-dependent Cox proportional hazard model.

Model	Variables	coef	exp (coef)	*P* value
Model I	Periodontal treatment experience (ref = Yes)			
	No	0.688	1.991	< 0.001
Model II	Periodontal treatment experience (ref = Yes)			
	No	0.602	1.826	< 0.001
	Systematic diseases (ref = No)			
	Ischemic heart diseases	0.339	1.404	< 0.001
	Cerebrovascular diseases	0.067	1.069	< 0.001
	Kidney diseases	1.177	3.247	< 0.001
	Diabetes mellitus	0.432	1.541	< 0.001
	Sex (ref = Male)			
	Female	− 0.632	0.531	< 0.001
	Age	0.051	1.052	< 0.001
	Health Insurance (ref = Health insurance holders)			
	Medical aid	0.139	1.150	< 0.001
	Residential areas (ref = metropolitan areas)			
	Small/medium-sized cities	− 0.032	0.968	< 0.001
	Rural areas	− 0.023	0.977	< 0.001

*Coef* coefficients, *ref* Reference group.

**Figure 2:**
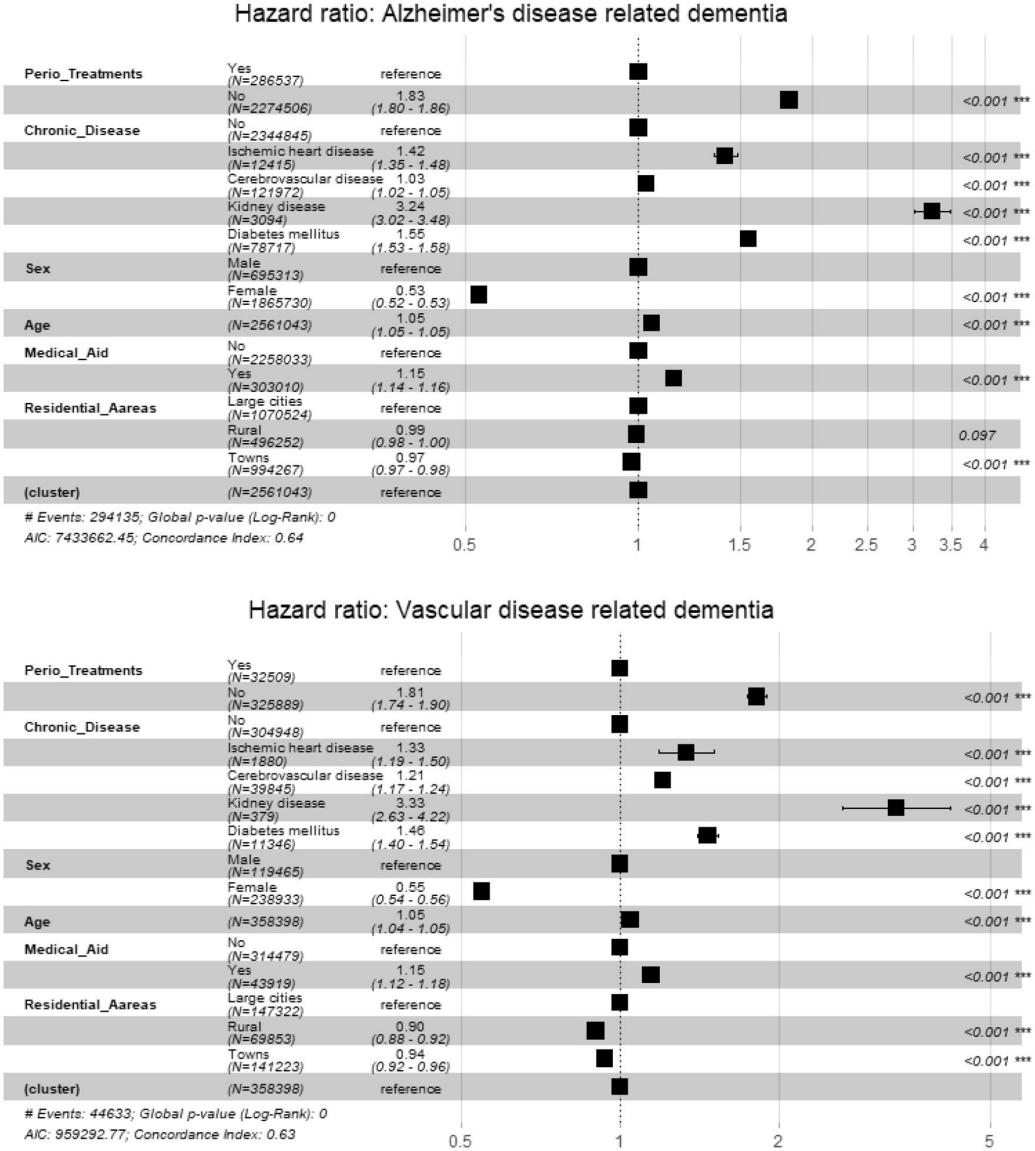
Forest plot for the time-dependent Cox proportional hazards model. (**a**) Alzheimer’s disease-related dementia and (**b**) vascular disease-related dementia.

To examine whether the association of independent variables with the dependent variable vary according to the type of dementia, the mortality risk of the independent variables was plotted after calculating the adjusted HR of the independent variables (Fig. 2). The influence of cerebrovascular disease and non-metropolitan residents was different in the vascular dementia group and the Alzheimer’s dementia group; the influence of the other variables was similar between the two groups.

The results of the time-dependent Cox proportional hazards models according to periodontal treatment classification for dementia are presented in Supplementary Table S1 online.

## Discussion

This study investigated the association between periodontal treatments and dementia-related mortality among 1.31 million patients with dementia over the age of 65 years who used dental services between 2002 and 2018. Periodontal care was associated with a reduced mortality risk in patients with dementia. Dementia-related mortality is related to the oral and systemic health statuses. This study revealed that periodontal treatments might contribute to reducing dementia-associated mortality even after adjusting for the major chronic disease status of patients with dementia.

Previous studies have consistently reported a relationship between dementia and oral health-related diseases. Participants with Alzheimer’s disease tend to have a smaller number of teeth; higher rate of decayed, missing, and filled teeth; and lower oral health index than those of the control group^16–18^. The incidence of denture-related stomatitis is high in patients with Alzheimer’s disease, which is reportedly attributed to cognitive decline^19^.

Previous studies reporting the relationship between dementia and periodontal disease can be divided into those using epidemiological parameters^11,12,17,20^ and microbiological approaches^10,13,15,21–23^. Patients with dementia have a poor community periodontal index^20^, poor plaque index, loss of clinical attachment^23^, and bleeding on probing levels^17,21^. The incidence and mortality of Alzheimer’s disease in older individuals are related to periodontal pathogens^15^. In addition, candidiasis, stomatitis, and reduced salivary flow have been reported in patients with dementia^21^. Sharma et al.^24^ suggested that periodontitis might increase mortality owing to the increased burden of systemic inflammation in patients with chronic kidney disease. A recent study reported that a lower estimated glomerular filtration rate is correlated with a higher incidence rate of dementia^25^. Nadim et al.^22^ predicted that reducing the prevalence of periodontal disease by 20% could reduce the occurrence dementia-related mortality in 850,000 individuals (630,000–1,420,000) worldwide. Other previous studies have also demonstrated a clear relationship between dementia and oral health status.

In a study examining dementia and death, Connors et al.^26^ reported that the HR for dementia increased with the increase in its severity. A cohort study with a 13-years follow-up period reported that nearly one-third of deaths among people aged ≥ 85 years might be due to dementia^1^. A population-based study reported that the HR of mortality risk in the incident dementia group was higher than that in the prevalent dementia group^27^. Mortality in patients with dementia increases with the increase in the incidence of comorbidities^28^, and dementia-associated mortality is affected by disease progression^29^.

Microbial agents, including periodontal pathogens, are important factors in Alzheimer’s disease, and the association of periodontal disease with Alzheimer’s disease-related mortality has been reported. Beydoun et al.^15^ reported that the incidence and mortality of Alzheimer’s disease in people aged over 65 years were associated with attachment loss, probing pocket depth, and pathogen immunoglobulin G as periodontal markers. The findings of the study by Beydoun et al.^15^ may provide evidence for the intervention of periodontal treatment in delaying death in patients with dementia, which was the aim of this study.

Although the proportion of females with dementia in this study was approximately 2.2 times higher than that of males, the risk of death was higher in males; this is similar to the results of several previous studies^1,26,28^. Taudorf et al.^28^ confirmed that the proportion of females was higher than that of males; however, the mortality rate was higher for males in all age groups in a 5-years-old unit. In a study using cohort data, the proportion of females in the dementia group was also high; however, the HR of males was 2.4 times higher than that of females^1^. This finding was explained by the fact that females had a longer initial period of low dementia intensity than males, and the progression of dementia was slower in females than in males^30^.

In 2021, medical aid was delivered for approximately 7.4% of the population aged ≥ 65 years in South Korea^31^. Twelve percent of the study participants were identified as having received medical aid, which is higher than the average representative figure. Furthermore, the risk of death in the patients with dementia who received medical aid was 1.15 times higher, which is consistent with the findings of previous studies^32,33^. Among patients with dementia, lower individual socioeconomic position (SEP) was associated with a higher HR for dementia mortality compared to those with higher SEP^33^, and low household income was the strongest predictor of death^34^. These results imply that dementia occurs more frequently in economically vulnerable groups and influences the mortality rate.

This study had several strengths as it contributes to the knowledge of the association between oral disease interventions and dementia-associated mortality. Based on the results, it might be argued that periodontal treatment decreases the mortality rate among patients with dementia. Moreover, this study used cohort data for a follow-up duration of 17 years using the National Health Insurance (NHI) database.

Nevertheless, this study also had some limitations. First, it is difficult to obtain information on the details of out-of-bound NHI benefits because the data only included details of dental services that entailed NHI-covered treatments; however, the NHI covers the service of nearly all periodontal treatments. In addition, the study population only included patients with dementia who visited dental hospitals and clinics between 2002 and 2018, and people with dementia who did not visit a dentist were excluded. However, as our study had a large sample size, encompassing all seniors who visited a dentist during the study period, we believe that the impact of sampling bias on the results will be negligible. Finally, this study was conducted using a limited set of variables from NHI claims data in South Korea, which limits its ability to capture a wide range of variables such as demographic characteristics that entail educational level, ethnicity, cultural diversity, lifestyle choices, and genetic predispositions.

In conclusion, this study revealed that the risk of death in patients with dementia who did not receive periodontal treatment was higher than that in patients who received periodontal treatment, despite the fact that the demographic variables and systematic chronic diseases were adjusted using cohort data for 17 years. Our findings might provide evidence for the need for dental intervention to maintain or improve the health of older individuals with dementia in South Korea. This could be implemented by expanding the scope of dental services in the elderly care facilities and developing a curriculum for oral hygiene education for care providers.

## Methods

### Ethics declarations

The study design was approved by the Institutional Review Board of Wonkwang University, South Korea (Approval Number: WKIRB–201911-SB-082). All the methods were carried out in accordance with the relevant guidelines and regulations. The need for informed consent was waived by the ethics Institutional Review Board of Wonkwang University because of the retrospective nature of the study. The data used in this study were anonymously provided by the National Health Insurance Service.

### Study design and participants

South Korea operates the National Health Insurance Service in the form of social insurance, and all citizens are mandated to be enrolled in this service. This study used the NHI claims data linked to the death registry of Statistics in Korea. The inclusion criteria for our study were patients (1) aged ≥ 65 years, (2) diagnosed with dementia from a population of patients who received inpatient and outpatient treatments for dental diseases for 17 years between 2002 and 2018, and (3) received oral care after being diagnosed with dementia. The data were organized in the form of retrospective cohort study data, and the main diagnoses were limited to codes G30 and F00 (Alzheimer’s disease) and F01 (vascular dementia) according to the International Classification of Diseases, Tenth Revision^35^. Periodontal treatment was classified according to the Korean Standard Terminology of Medicine (KOSTOM)^36^ based on the actual treatment history of the study participants. The dental procedure classification to define periodontal treatment, we extracted Scaling, Root planning, Subgingival curettage, Periodontal flap surgery (Scaling: U2232, U2233, Root Planning: U2240, Subgingival curettage: U1010, Periodontal flap surgery: U1020–U1090, U1100, U1110) from the KOSTOM standard practice codes. As non-surgical treatments, scaling and root planning were combined into SRP, and the SRP condition was defined as receiving scaling or root planning.

This study used claims data provided by the National Health Insurance (NHI), we used periodontal treatment as the actual treatment code for the patients. This is attributed to the fact that the claims data do not provide a clear baseline of the patients’ oral condition at that time. Therefore, this study focused on analyzing the association between periodontal treatment and mortality in patients with dementia as an effect of continuous or comprehensive periodontal treatment, rather than the association between individual periodontal treatment and mortality in patients with dementia.

Patients who had received periodontal treatment (SRP, subgingival curettage, periodontal flap surgery) after a confirmed history of dementia were selected as the experimental group, whereas patients with dementia who had not received any of the aforementioned periodontal treatments were selected as the control group. The final analysis included 1,131,406 participants.

### Variables

The dependent variable was death. Sex, age, medical aid, and residential area were used as demographic variables of the study participants. Age was used as a continuous variable in the units of 1 year. Residential areas, which are a proxy variable for a similar distribution of dental resources, were divided into metropolitan areas, small and medium-sized cities, and rural areas, similar to the distribution of medical resources. In South Korea, social health insurance is compulsory for all citizens, and premiums are charged according to the income level. The NHI claims data contained various health insurance premiums; however, the insurance premium variable was excluded from the final analysis due to the multicollinearity effect of the low-income class and medical aid variable in the preliminary analysis. Receiving periodontal treatment was divided into “Yes” and “No”. To adjust for the general health status, chronic diseases affecting oral health^32,37^ were classified into ischemic heart disease, cerebrovascular disease, diabetes mellitus, and renal disease based on the main diagnosis.

### Statistical analyses

Baseline characteristics of the participants stratified by dementia type were first compared using the chi-squared test for categorical variables and independent *t*-test for continuous variables. Next, we used longitudinally organized cohort data to examine whether periodontal disease treatment was related with the survival rate of patients with dementia. The Cox proportional hazards regression model applied to survival analysis cannot reflect the change in covariate values according to the change in follow-up time because of the proportional hazard assumption. The Cox proportional hazards model with time-dependent covariates may have a bias in the correlation between independent and outcome variables^38–40^. In this case, a time-dependent Cox proportional hazard model is recommended, as it can reflect the status of the time-dependent covariates. Since the chronic diseases in this study were classified based on the main diagnosis at the time the patient visited the medical institution for a specific medical symptom, there is a possibility that the main diagnosis of patients with multiple chronic diseases may vary at each visit or change over time. A violation of the proportional hazard assumption was confirmed using Kaplan–Meier plots. Model 1 for time-dependent Cox proportional hazard regression only included the periodontal treatment variable, whereas Model 2 included other covariates as well as periodontal treatments. All statistical analyses ware performed using R (version 4.0.1; R Foundation for Statistical Computing, Vienna, Austria). A *P* value < 0.05 was used to indicate statistical significance.

### Supplementary Information

Supplementary Table S1.

## Data Availability

The data that support the findings of this study are available from National Health Insurance Service but restrictions apply to the availability of these data, which were used under license for the current study, and so are not publicly available. Data are however available from the authors upon reasonable request and with permission of National Health Insurance Service.
