# Supplementary material for: Association of periodontal disease treatment with mortality in patients with dementia: a population-based retrospective cohort study (2002–2018)
Source: Sci Rep. 2024 Mar 4;14:5243. doi: 10.1038/s41598-024-55272-6 (PMC10912191; doi:10.1038/s41598-024-55272-6)
Supplement: Supplementary file 1 — Supplementary Table S1. [file 41598_2024_55272_MOESM1_ESM.docx]

**Supplementary Tables**

**Supplementary Table S1.** The time-dependent Cox proportional hazard model for dementia.

Supplementary Table S1. The time-dependent Cox proportional hazard model for dementia.

| Model | Variables | |  |  | coef. | exp (coef) | *p*-value |
| --- | --- | --- | --- | --- | --- | --- | --- |
| Model I | Periodontal treatment classification (ref=No) | | | | | | |
|  |  | SRP |  |  | -0.624 | 0.535 | < 0.001 |
|  |  | Subgingival curettage |  |  | -1.028 | 0.358 | < 0.001 |
|  |  | Periodontal flap surgery |  |  | -1.051 | 0.350 | < 0.001 |
| Model II | Periodontal treatment classification (ref=No) | | | | |  |  |
|  |  | SRP |  |  | -0.545 | 0.579 | < 0.001 |
|  |  | Subgingival curettage |  |  | -0.903 | 0.405 | < 0.001 |
|  |  | Periodontal flap surgery |  |  | -0.954 | 0.385 | < 0.001 |
|  | Systematic Diseases (ref=No) | | | |  | | |
|  |  | Ischemic heart diseases | | | 0.340 | 1.405 | < 0.001 |
|  |  | Cerebrovascular diseases | | | 0.067 | 1.069 | < 0.001 |
|  |  | Kidney diseases | | | 1.178 | 3.249 | < 0.001 |
|  |  | Diabetes mellitus | | | 0.433 | 1.541 | < 0.001 |
|  | Sex (ref=Male) | | | |  |  |  |
|  |  | Female | | | -0.632 | 0.531 | < 0.001 |
|  | Age | | |  | 0.051 | 1.052 | < 0.001 |
|  | Health Insurance (ref= Health insurance holders) | | | | | | |
|  |  | Medical aid |  |  | 0.139 | 1.149 | < 0.001 |
|  | Residential areas (ref=metropolitan areas) | | |  |  |  |  |
|  |  | Small/medium-sized cities | | | -0.032 | 0.968 | < 0.001 |
|  |  | Rural areas | | | -0.023 | 0.977 | < 0.001 |

*Coef,* Coefficients; *ref,* Reference group; *SRP,* Scaling or Root planning
